# Synthesis and Characterization of a Propolis-Containing Cellulosic Textile Functionalized with a Crosslinked Agar/κ-Carrageenan Biopolymer Network: Structure–Property Relationships and In Vitro Biological Responses

**DOI:** 10.3390/polym18212794

**Published:** 2026-11-08

**Authors:** Yassine El-Ghoul, Sami M. Alharbi, Suliman S. Aba-alkhayl, Bechir Hammami

**Affiliations:** Department of Chemistry, College of Science, Qassim University, Buraidah 51452, Saudi Arabia; sam.alharbi@qu.edu.sa (S.M.A.); chemist.suliman@gmail.com (S.S.A.-a.); b.hammami@qu.edu.sa (B.H.)

**Keywords:** agar, κ-carrageenan, cellulose functionalization, poly(acrylic acid), propolis, crosslinked biopolymer network, antimicrobial activity, cell viability

## Abstract

The development of multifunctional wound dressings based on bioactive polymers offers an opportunity to combine moisture management, structural stability, and biological functionality within a single material. However, the integration of crosslinked agar/κ-carrageenan coatings, a poly(acrylic acid)/sodium hypophosphite (PAA/SHP) crosslinking system, and propolis on cellulosic textile substrates, together with the relationships between material properties and in vitro biological responses, warrants further investigation. In this study, a functionalized cellulosic textile incorporating agar, κ-carrageenan, PAA, SHP, and propolis (PS) was prepared and characterized. The curing conditions of 140 °C for 15 min were selected based on previous work and adopted in the present study. The resulting materials were characterized by ATR-FTIR spectroscopy, scanning electron microscopy (SEM), swelling measurements, and tensile testing. The functionalized textiles exhibited a mass gain of 23.03% and a swelling ratio of 630%, indicating substantial material incorporation and water uptake. The wet breaking force reached 4.89 N, demonstrating measurable mechanical performance under hydrated conditions. ATR-FTIR findings were consistent with chemical interactions associated with the proposed PAA-mediated crosslinking mechanism, while SEM revealed morphological features consistent with the formation of a polymeric coating on the cellulose fibers. The complete formulation exhibited an apparent relative HepG2 MTT response of 132.5% after 72 h, indicating metabolic activity above that of the control under the tested conditions. The propolis-containing materials also exhibited antibacterial activity against the Gram-positive bacterium *Staphylococcus aureus* and the Gram-negative bacterium *Escherichia coli*, with inhibition zones of 39.2 mm and 29.3 mm, respectively, and demonstrated antifungal activity against *Candida albicans*. Further evaluation using MCF-7 breast cancer cells and non-tumorigenic MCF10A breast epithelial cells revealed differential cellular viability responses. MCF-7 cell viability decreased to 25.4% and 17.2% after 24 and 48 h, respectively, under the tested conditions. These findings highlight the material’s water-absorption capacity, mechanical performance, antimicrobial activity, and differential cell-based responses. The novelty of this study lies in integrating these components within a cellulosic textile and evaluating its physicochemical, mechanical, antimicrobial, and cellular responses. Overall, the results support further investigation of the prepared material as a candidate bioactive wound dressing. Nevertheless, additional studies are required to establish the nature and extent of covalent grafting, quantify propolis loading and release, and assess compatibility with wound-relevant cells, particularly human dermal fibroblasts and keratinocytes.

## 1. Introduction

Chronic and acute wounds are still a major global public health problem, with long healing durations, high risk of infection, and substantial impact on patients’ quality of life [1,2,3]. Effective wound management needs the use of products that are capable of maintaining a moist environment, preserving the wound bed, reducing microbial contamination, and encouraging tissue restoration [4,5,6,7].

Although various wound-care treatments are available, many conventional dressings remain passive materials that primarily act as physical barriers, with limited intrinsic biological activity [8,9,10,11]. Such dressings may be insufficient for managing chronic or infected wounds, which can require effective moisture management, antimicrobial protection, and support for tissue repair. Accordingly, an ideal wound dressing should combine biocompatibility, breathability, appropriate fluid absorption and retention, and antimicrobial activity, while supporting the physiological processes involved in wound healing [12,13,14,15,16].

To address the limitations of passive dressings, increasing attention has been directed toward functionalized polymer-based materials [17,18,19,20,21,22]. Various approaches have been investigated to develop wound dressings incorporating natural or synthetic polymers and bioactive compounds. For example, cellulosic dressings functionalized with polysaccharides, honey, silver nanoparticles, or plant extracts have demonstrated promising antimicrobial or wound-healing-related properties [23,24,25,26,27,28]. Alginate- and hyaluronic acid-based systems incorporating curcumin, aloe vera, or antimicrobial peptides have also been investigated for their potential antioxidant, anti-inflammatory, and antimicrobial effects [29,30,31,32]. In addition, antibiotics, essential oils, and metallic nanoparticles have been incorporated into electrospun nanofibrous materials based on cellulose, polycaprolactone (PCL), poly(vinyl alcohol) (PVA), gelatin, and other natural polymers to provide functional activity and, in some cases, controlled release of active compounds [33,34,35,36,37,38]. Collectively, these studies highlight the potential of multifunctional dressings that integrate the structural properties of polymeric materials with selected biological activities.

Within this context, chemical functionalization and crosslinking are important strategies for developing polymer-based wound-dressing materials with improved structural stability and application-specific properties [39,40,41]. These approaches can modify polymer networks, improve coating retention, and influence moisture management and the incorporation of bioactive compounds. Natural polysaccharides such as agar and κ-carrageenan are attractive components because of their hydrophilicity, film- and gel-forming capabilities, and potential suitability for biomedical materials. However, their performance in textile-based dressings may depend on the stability of the deposited layer and its interaction with the underlying substrate. Crosslinking and the incorporation of selected bioactive compounds therefore offer potential routes to tailoring the properties of these materials [42,43,44].

Despite their attractive functional properties, polysaccharide-based coatings may require additional stabilization to maintain their integrity during aqueous exposure. In the present study, poly(acrylic acid) (PAA) was selected as a crosslinking component in combination with sodium hypophosphite (SHP) as a catalyst. PAA contains carboxyl groups that, under suitable thermal curing conditions, may react with hydroxyl groups in cellulose and polysaccharides, including agar and κ-carrageenan. SHP is commonly used to promote carboxylic-acid-mediated esterification in cellulose-based systems, potentially facilitating the formation of covalent linkages within the treated material [45]. Such reactions may improve coating retention and contribute to the structural integrity of the resulting material during aqueous exposure. Nevertheless, the extent of crosslinking and the resulting material properties depend on the formulation and processing conditions. Furthermore, residual reagents must be considered when evaluating the suitability of the material for biomedical applications. Accordingly, the effects of the PAA/SHP treatment require experimental assessment rather than being assumed from the proposed reaction mechanism alone.

Propolis is a natural resinous material containing phenolic compounds and flavonoids that have been associated with antioxidant, antimicrobial, and anti-inflammatory activities [46,47,48]. These properties have prompted interest in its incorporation into polymer-based materials intended for wound-care applications. Combining propolis with a polysaccharide-containing coating on a cellulosic textile may provide a means of integrating the structural functions of the polymeric components with the potential biological activity of the extract. However, the performance of such a material depends on its formulation, the incorporation and retention of propolis-derived compounds, and the biological endpoints used for evaluation.

Against this background, the present study aimed to prepare and characterize a woven cotton-gauze substrate treated with a PAA/SHP-containing agar/κ-carrageenan formulation incorporating propolis extract. The novelty of this study lies in the integration of a cellulosic textile substrate, an agar/κ-carrageenan polysaccharide coating, a PAA/SHP crosslinking system, and propolis extract within a single textile-based material, together with the combined evaluation of its physicochemical, swelling, mechanical, and in vitro biological properties. The effects of selected processing conditions on the measured mass-gain response were investigated, and the resulting material was characterized in terms of its chemical features, morphology, swelling behavior, and mechanical properties. Its antimicrobial activity and cell-based responses were also evaluated under the specified in vitro assay conditions. By examining these properties together, this study sought to establish formulation-associated structure–property relationships and assess the potential of the treated textile as a candidate wound-dressing material. The findings are interpreted in light of the characterization methods and biological models employed, recognizing that further studies are needed to establish the extent of covalent crosslinking, propolis loading and release, and suitability for wound-related applications.

## 2. Materials and Methods

### 2.1. Materials

The substrate consisted of sterile, bleached, multilayer woven cotton gauze supplied by Adhe-Els Laboratory (Kalâa Kebira, Sousse, Tunisia) for research purposes. The textile had a thread density of 17 yarns cm^−1^, an areal weight of 180 g m^−2^, and a thickness of approximately 1.22 mm.

κ-Carrageenan (CAR), agar (AGR), poly(acrylic acid) (PAA), and sodium dihydrogen hypophosphite (SHP) were purchased from Aldrich Chemical Co. (Milwaukee, WI, USA) and utilized without further purification. White propolis powder was collected in 2025 in the Nord region, Tunisia. The crude propolis (PS) was ground up with a mortar and pestle and then promptly frozen in liquid nitrogen to prevent oxidation and loss of volatile components. The treated samples were then stored in airtight polyethylene bags at temperatures below −20 °C until further use. Textile functionalization was carried out by a laboratory padder and a hot-air curing oven following a standard pad–dry–cure process.

### 2.2. Functionalization of Woven Cotton Gauze with CAR/AGR/PAA/SHP and Propolis

The dressing samples were first washed in hot water to eliminate contaminants and to increase the uptake of polymer. After drying (1 h at 104 °C), functionalization was carried out by a pad–dry–cure procedure modified from earlier reports [49,50]. The fabrics were impregnated with the grafting bath and then put through a padding machine for even distribution of the solution.

Cellulosic textile specimens measuring 4 × 4 cm were prepared for the physicochemical assessments. Separate, larger specimens were prepared for tensile testing, as described in Section 2.5. Both specimen types were functionalized with formulation C under identical processing conditions using a laboratory padder (EHP 350, Roaches International Ltd., Birstall, United Kingdom). The impregnation formulation consisted of agar (40 g L^−1^), κ-carrageenan (60 g L^−1^), poly(acrylic acid) (PAA, 80 g L^−1^), and sodium hypophosphite (SHP, 10 g L^−1^) dissolved in distilled water. After thorough stirring to ensure complete dissolution, an ethanolic propolis extract was added at a final volume fraction of 10% (*v*/*v*) in the impregnation bath. The stated 10% (*v*/*v*) refers to the volume fraction of the ethanolic propolis extract added to the impregnation bath. It does not represent a measured propolis concentration in the finished textile. No quantitative determination of propolis loading, retained phenolic content, loading efficiency, or release was performed. The pH of the resulting formulation was adjusted to 3. This prepared initial solution will be called C. The textile was passed through the padding rollers twice at a roller speed of 2 m min^−1^ and a squeezing pressure of 2 bar to achieve a consistent wet pick-up of approximately 100%. Following impregnation, excess solution was removed by roller squeezing, and the treated samples were dried at 104 °C for 30 min to eliminate residual moisture before the subsequent curing step.

The polymerization and crosslinking of the polysaccharide-PAA system on the cellulose fibers were then promoted by thermal curing at specific temperatures and periods. The cured dressing was then treated with an alkaline solution of ammonium hydrogen phosphate at 2% (*w*/*v*) to eliminate ungrafted polymer and to improve the handling of the finished dressing. Thereafter, the samples were subjected to severe washings with hot water and finally dried for 1 h at 104 °C.

The mass gain was calculated using Equation (1):

(1) $$\mathrm{Mass}\;\mathrm{gain}\;(\%)=\frac{\left[\mathrm{mf}-\mathrm{mi}\right]}{\mathrm{mi}}\;\times \;100$$

where *mi* is the initial dry mass of the substrate, and *mf* corresponds to the final dry mass after treatment and the specified washing procedure. All measurements were performed in triplicate. Mass gain represents the net dry mass retained after treatment and washing. It is not a component-specific measurement and cannot be used to quantify retained propolis or the extent of covalent grafting.

The effect of the initial polymer concentration on grafting mass gain and mechanical properties was evaluated using five concentration levels: untreated, C/4, C/2, 3C/4, and C. Here, C denotes the initial concentration of the complete impregnating solution containing κ-carrageenan, agar, poly(acrylic acid) (PAA) as the crosslinking agent, and propolis. The corresponding grafting rates were 0%, 5.77%, 11.52%, 17.31%, and 23.03%, respectively (Table 1). All functionalized samples were subjected to the same curing conditions (140 °C for 15 min), selected based on our previous optimization study, to investigate the effect of polymer concentration while maintaining constant thermal treatment.

### 2.3. ATR-FTIR Analysis

Chemical modifications associated with the grafting process were examined by attenuated total reflection Fourier-transform infrared spectroscopy (ATR-FTIR). Spectra were recorded on an Agilent FT-IR spectrometer (Agilent Technologies, Inc., Santa Clara, CA, USA) equipped with a GladiATR accessory (PIKE Technologies, Inc., Madison, WI, USA) and obtained with a configuration of 32 scans and a resolution of 4 cm^−1^, covering a range from 4000 to 400 cm^−1^.

### 2.4. Swelling Behavior

The swelling behavior of untreated and functionalized dressings was evaluated gravimetrically in triplicate (*n* = 3). Specimens measuring 4 × 4 cm were dried to constant mass and weighed to determine their initial dry mass (*mi*), which was approximately 0.288 g for the untreated textile. Each specimen was then immersed in 100 mL of distilled water at room temperature for predefined time intervals. At each time point, specimens were removed from the water, gently blotted with absorbent paper to remove excess surface water, and immediately weighed (*mf*). The swelling ratio was calculated using the following equation:

(2) $$\%-\mathrm{SR}=\frac{\left(\mathrm{mf}-\mathrm{mi}\right)}{\mathrm{mi}}\;\times \;100$$

where *mf* and *mi* represent the initial dry weight and swollen weight, respectively.

### 2.5. Mechanical Properties (Tensile Testing)

The mechanical properties of the untreated and functionalized textiles were evaluated by tensile testing in accordance with NF EN ISO 13934-1 [51]. Separate textile strips, distinct from the 4 × 4 cm specimens used for physicochemical assessments, were prepared with a width of 50 mm and tested at an initial gauge length of 200 mm. Functionalized strips underwent the corresponding impregnation, curing, washing, and drying procedures before testing; untreated strips served as comparators. Before testing, the dry specimens were conditioned at 25 °C and 65% relative humidity (RH) for at least 48 h. Tensile tests were performed using a 2/M tensile-testing machine (Adamel Lhomargy, Paris, France), equipped with a 10 kN load cell and clamps featuring a constant-pressure gripping system. The crosshead speed was fixed at 100 mm min^−1^. Ten specimen replicates were tested for each condition, and results were expressed as mean ± standard deviation.

### 2.6. Scanning Electron Microscopy (SEM)

The surface morphology of the untreated and functionalized wound-dressing samples was examined using a scanning electron microscope (SEM; JSM-5400LV, JEOL, Akishima, Japan). Micrographs were acquired at an accelerating voltage of 10 kV, with magnification adjusted as required to visualize the surface features and assess changes associated with textile functionalization. Before examination, the samples were sputter-coated with a thin layer of gold to improve surface electrical conductivity and minimize charging effects during imaging. Micrographs were used to compare the structural features of the untreated and grafted dressings and to evaluate the distribution of the biopolymer coating.

### 2.7. Biological Evaluation

HepG2-based MTT and adhesion-associated assays were used to investigate in vitro cellular responses to the untreated and functionalized textiles. These experiments were not designed to establish wound-healing efficacy, comprehensive biocompatibility, or absence of toxicity.

#### 2.7.1. Cell Viability Assessment (MTT Assay)

The cytocompatibility responses of the virgin and functionalized dressings were evaluated using the MTT colorimetric test, the most common method for measuring cell metabolic activity. HepG2 cells were cultivated under ideal conditions (37 °C, 5% CO_2_ and 95% relative humidity) until the required confluence was reached. Textile disks (11 mm in diameter) were sterilized under UV radiation for 30 min on each side and pre-soaked in complete culture medium for 1 h before testing. Samples were then placed in direct contact with the cell monolayers in 24-well plates and incubated for 24, 48, and 72 h. After each incubation period, the culture medium was replaced with fresh medium containing the MTT reagent, and the cells were incubated for an additional 4 h. Metabolically active cells reduced the tetrazolium salt to purple formazan crystals. After incubation, the medium was carefully aspirated, and the formazan crystals were completely dissolved in DMSO. The absorbance was measured at 570 nm on a microplate reader. Cell viability (%) was determined by comparing the absorbance of cells treated with samples to that of untreated control cells, deemed 100% viable. This assay was used to quantitatively assess cellular metabolic activity and evaluate the in vitro cytocompatibility responses of the developed wound dressings toward the selected cell lines. Three separate experiments were carried out.

#### 2.7.2. Cell Adhesion

Cell adhesion onto functionalized and non-functionalized cellulosic dressings was investigated with the epithelial HepG2 cell line. Scaffold disks were inserted into 24-well plates and seeded with 4 × 10^4^ cells per sample in full culture media. Adhesion kinetics were measured at 30, 60, and 120 min of incubation at 37 °C. At each time point, the samples were gently washed in PBS to remove non-adherent cells. Then, 300 µL of p-nitrophenyl phosphate (p-NPP) substrate solution was added, and the alkaline phosphatase activity associated with adherent cells was quantified. The plates were incubated for 3 h, and the reaction was halted enzymatically by adding 150 µL of 1 N NaOH. p-nitrophenol release was monitored spectrophotometrically at 405 nm using an Apollo LB911™ microplate reader (Apollo 1 LB 911, Berthold Technologies GmbH & Co. KG, Bad Wildbad, Germany). Absorbance values, which are proportional to the amount of adhering cells, were measured in ten replicates for each condition to ensure statistical reliability.

### 2.8. Antimicrobial Activity by Agar Diffusion Assay

The antimicrobial activity of the untreated and functionalized dressings was evaluated using an agar diffusion method based on the Kirby–Bauer disk diffusion procedure [52]. The bacterial strains included *Staphylococcus aureus* (ATCC 25923) and *Enterococcus faecalis* (ATCC 29212) as Gram-positive bacteria, and *Escherichia coli* (ATCC 35218) and *Pseudomonas aeruginosa* (ATCC 33787) as Gram-negative bacteria. The bacteria were cultured in nutrient broth for 24 h at 37 °C and subsequently transferred to nutrient agar and incubated for a further 24 h at 37 °C. A bacterial suspension with a final concentration of 10^6^ CFU/mL was prepared in physiological saline. An aliquot of 1 mL of the suspension was spread onto Mueller–Hinton (MH) agar plates (Becton, Dickinson and Company, Sparks, MD, USA), which were maintained at 37 °C for 30 min before the textile specimens were applied.

Textile disks measuring 11 mm in diameter were exposed to UV irradiation for 30 min on each side for surface decontamination and placed on the inoculated Mueller–Hinton agar surfaces. The plates were held at 4 °C for 2 h to facilitate diffusion of potentially active compounds into the agar, followed by incubation at 37 °C for 24 h. Antibacterial activity was assessed by measuring the diameter of the inhibition zone surrounding each disk. Untreated textile disks underwent the same UV treatment and assay conditions and served as material comparators. Complete sterilization throughout the textile structure was not independently validated.

Antifungal activity was evaluated against *Candida albicans* (ATCC 90028). The yeast was cultured in Sabouraud--Chloramphenicol broth for 24 h at 25 °C and subsequently transferred to Sabouraud–Chloramphenicol agar and incubated for 24 h at 37 °C. A fungal suspension with a final concentration of 10^6^ CFU/mL was prepared in physiological saline. Sterile textile disks (11 mm in diameter) were placed on the prepared agar plates, followed by incubation at 37 °C for 24 h. Antifungal activity was assessed by measuring the inhibition zone surrounding each specimen.

All antimicrobial assays were performed in triplicate. The activities of the untreated and functionalized dressings were compared based on the measured inhibition zones.

### 2.9. MTT Responses in MCF-7 and MCF10A Cells

The in vitro effects of the dressings on cancer cell viability and their responses in non-tumorigenic cells were assessed using MCF-7 human breast cancer cells and MCF-10A non-tumorigenic human breast epithelial cells. MCF-7 cells were cultured in Dulbecco’s Modified Eagle’s Medium (DMEM) supplemented with 10% fetal bovine serum (FBS), 1% penicillin–streptomycin, and 1% L-glutamine, whereas MCF-10A cells were maintained in Mammary Epithelial Growth Medium (MEGM; Lonza Walkersville, Inc., Walkersville, MD, USA). Both cell lines were incubated at 37 °C in a humidified atmosphere containing 5% CO_2_.

Textile disks (11 mm in diameter) were sterilized by exposure to UV radiation for 30 min on each side and pre-soaked in complete culture medium for 1 h before testing. Cells were seeded in 96-well plates at a density of 1 × 10^4^ cells per well and allowed to adhere for 24 h. The textile disks were then placed in direct contact with the cell monolayers. Following 24 and 48 h of exposure, cellular metabolic activity was assessed using the MTT assay, and absorbance was measured at 570 nm. Relative cell viability was calculated as the percentage of absorbance in the treated group relative to that of the corresponding untreated control.

### 2.10. Statistical Analysis

Data are presented as mean ± standard deviation, with replication specified separately for each dataset. Tensile and cell-adhesion measurements comprised ten specimen replicates per condition. MTT assays comprised three separate experiments, each containing three technical replicates per condition. Technical replicates were averaged within each experiment before calculating the overall mean and standard deviation and conducting statistical comparisons; thus, the independent experimental sample size for MTT analysis was *n* = 3. Observations at different time points were obtained from separate specimens; the same specimen was not measured repeatedly over time. One-way ANOVA followed by Tukey’s HSD post hoc test was used to compare experimental groups within each assessment time or testing condition, and *p* < 0.05 was considered statistically significant using Statistica version 10 (StatSoft, Inc., Tulsa, OK, USA).

## 3. Results

### 3.1. Grafting Evaluation

**Figure 1:**
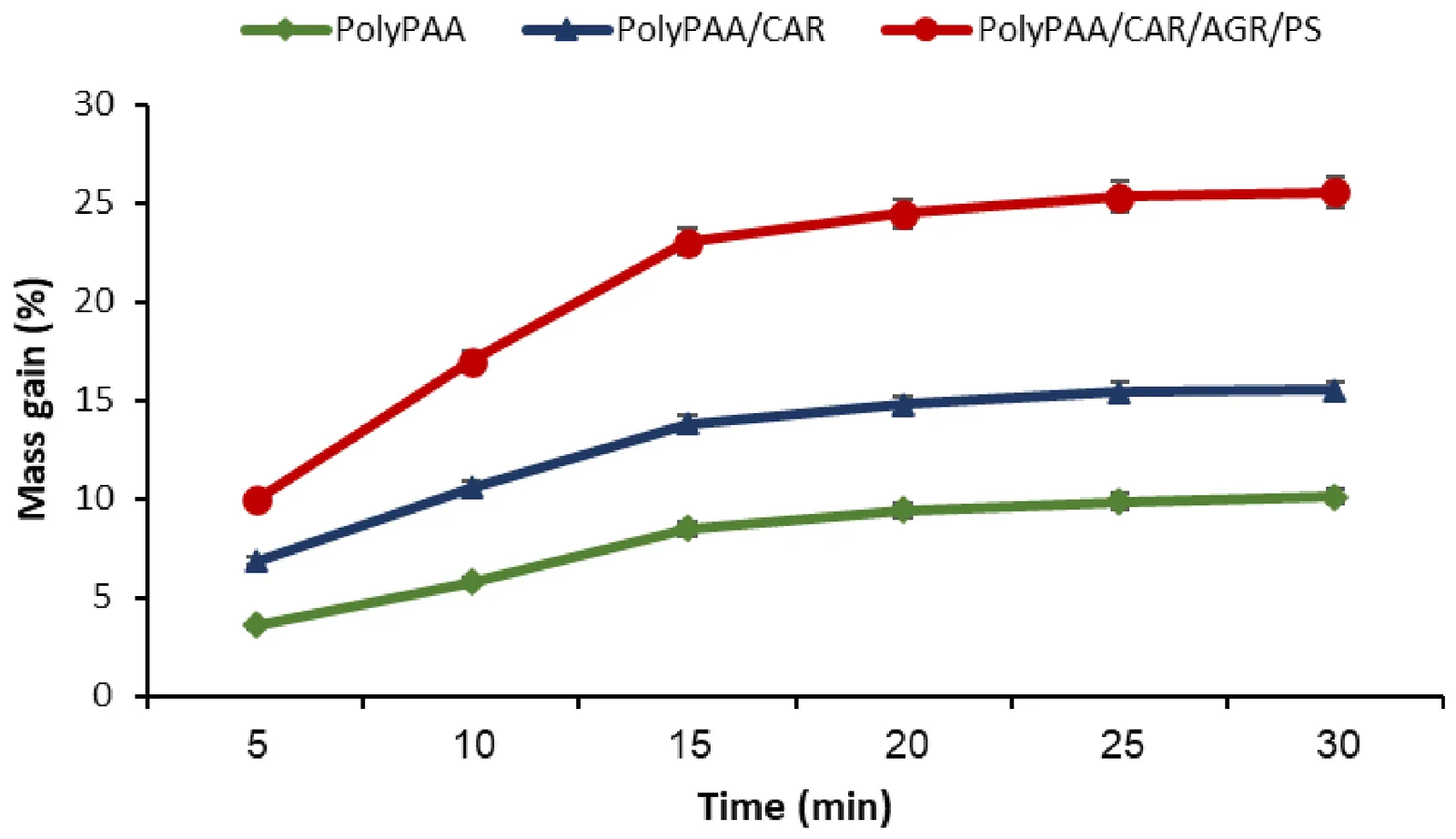
Effect of curing time on textile mass gain at a fixed temperature of 140 °C. All measurements were generated in the present study; the investigated processing range was informed by previous work [23,53].

**Figure 2:**
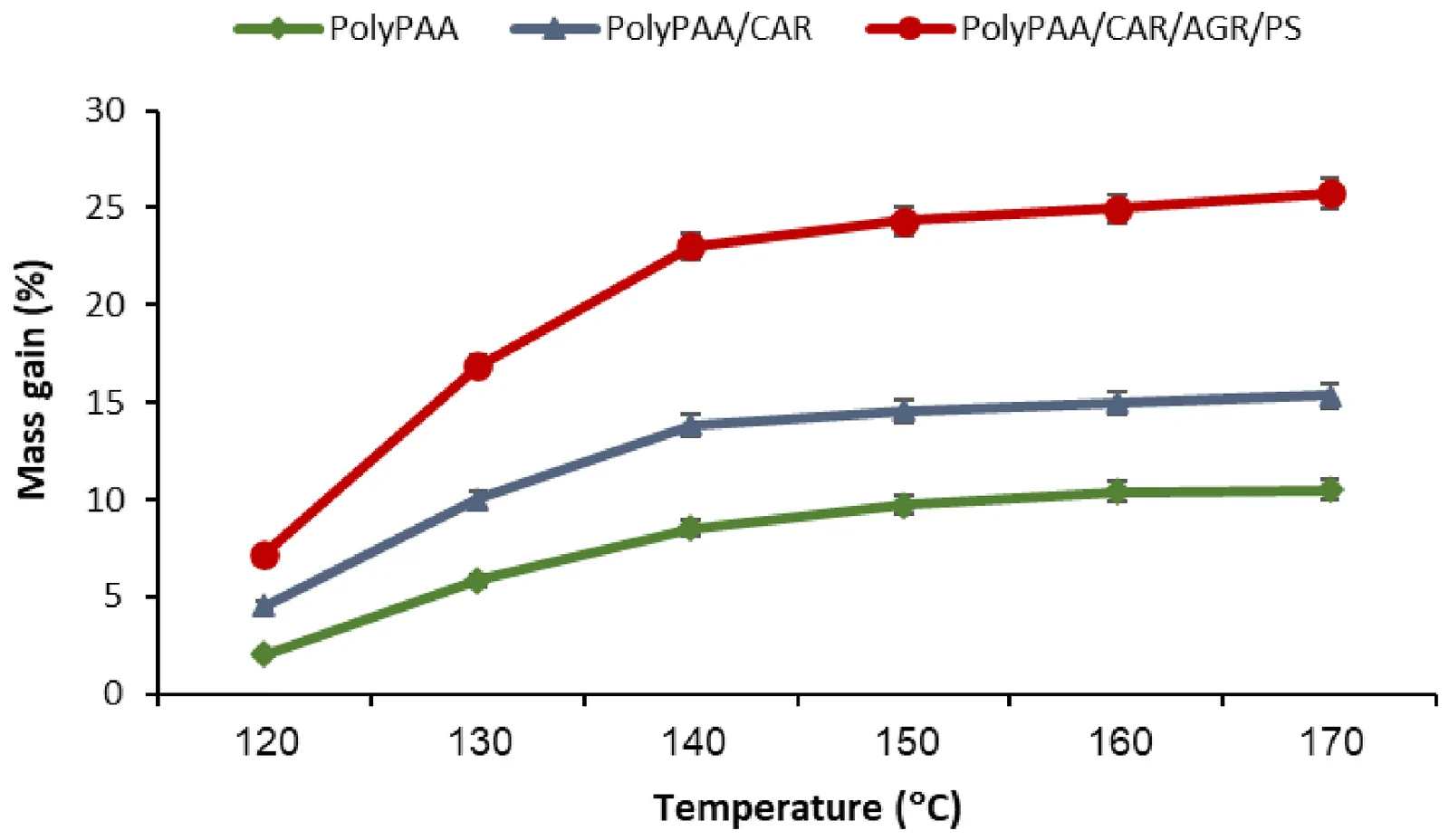
Effect of curing temperature on textile mass gain at a fixed curing time of 15 min. All measurements were generated in the present study; the investigated processing range was informed by previous work [23,53].

Our previous studies using response surface methodology (RSM) based on a Box–Behnken design (BBD) identified curing temperature and time as influential processing variables in related textile-functionalization systems [23,53]. These findings informed the selection of the variables and experimental ranges investigated here. All mass-gain measurements presented in Figure 1 and Figure 2 were newly generated in the present study for the formulations examined; no previously published datasets were reproduced. Curing time was investigated at a fixed temperature of 140 °C, whereas curing temperature was investigated at a fixed duration of 15 min. These experiments characterize the mass-gain response within the investigated ranges and do not constitute a new RSM or BBD optimization. The mass-gain response approached a plateau, indicating diminishing changes in retained mass rather than complete conversion of reactive sites or a quantified crosslinking density. Conditions of 140 °C for 15 min were selected for subsequent experiments near the onset of this plateau, where additional thermal exposure produced relatively small increases in mass gain. This selection also sought to limit unnecessary thermal exposure, since higher temperatures or longer curing times may adversely affect textile appearance, flexibility, and material stability; these potential effects were not independently quantified in the present study.

The grafting rate of the PAA/CAR/AGR/PS system was slightly higher than that of the PAA/CAR system. This difference may be associated with the incorporation of agar (AGR), which provides additional hydroxyl groups that may participate in interactions with the carboxyl groups of PAA during thermal curing. In polysaccharide esterification, primary hydroxyl groups at the C6 position are generally more accessible for esterification than the secondary hydroxyl groups at C2 and C3, although their relative reactivity depends on the polymer structure and reaction conditions [54,55]. The presence of agar may therefore contribute to the observed increase in grafting mass gain; however, this proposed mechanism requires further experimental confirmation.

**Figure 3:**
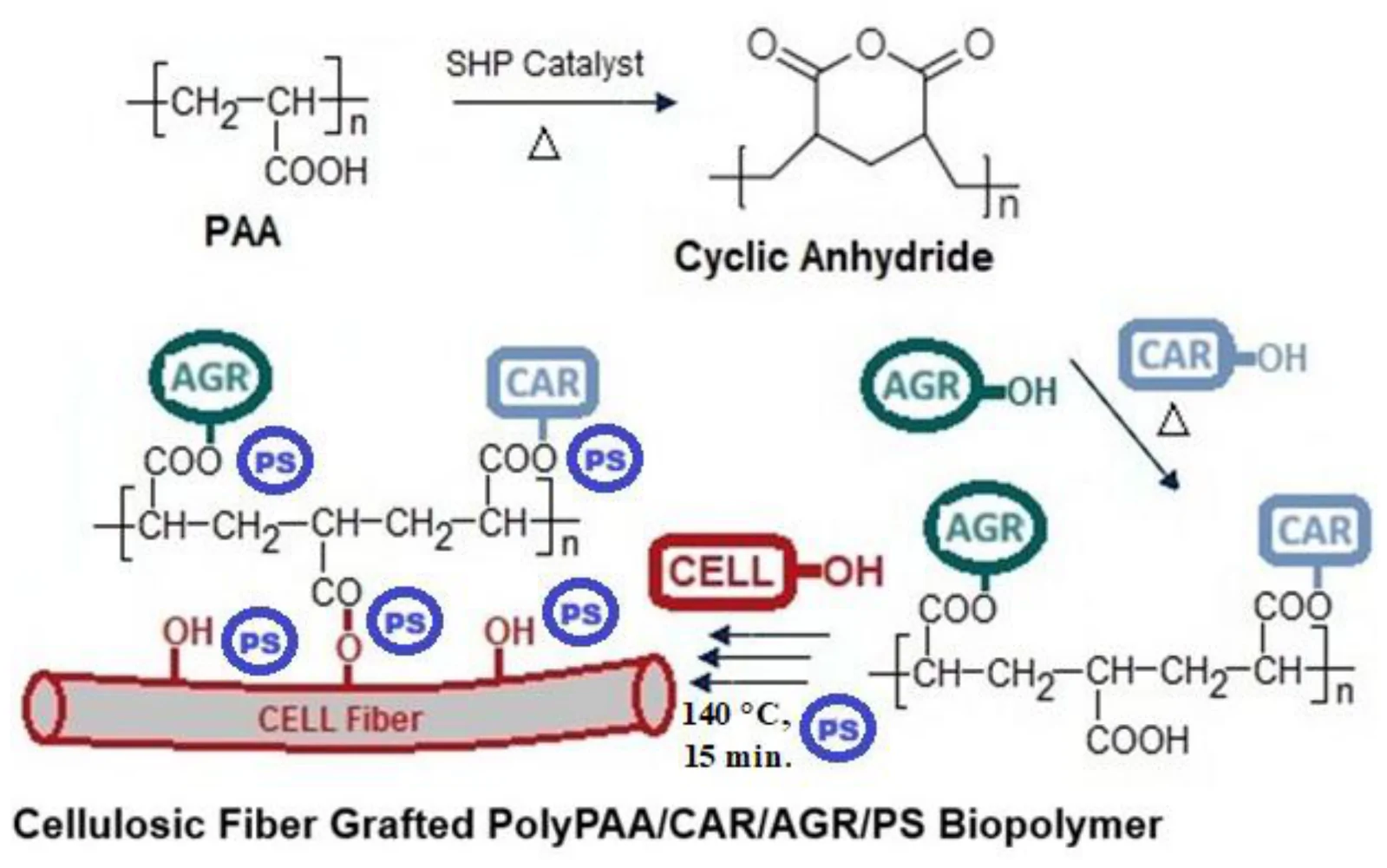
Proposed PAA-mediated esterification pathways involving cellulose and CAR/AGR hydroxyl groups. CELL: cellulose, CAR: carrageenan, AGR: agar, PS: propolis. The scheme illustrates possible reactions rather than experimentally verified bond formation or component connectivity.

Figure 3 presents a proposed PAA-mediated esterification pathway involving carboxyl groups of PAA and hydroxyl groups of cellulose and the polysaccharides. Such reactions may contribute to coating stabilization during thermal treatment. However, the present measurements do not independently identify the resulting covalent bonds, quantify their extent, or distinguish their contribution from hydrogen bonding, physical entrapment, and other interactions. Covalent attachment or quantitative retention of propolis is not demonstrated.

### 3.2. Swelling Behavior

**Figure 4:**
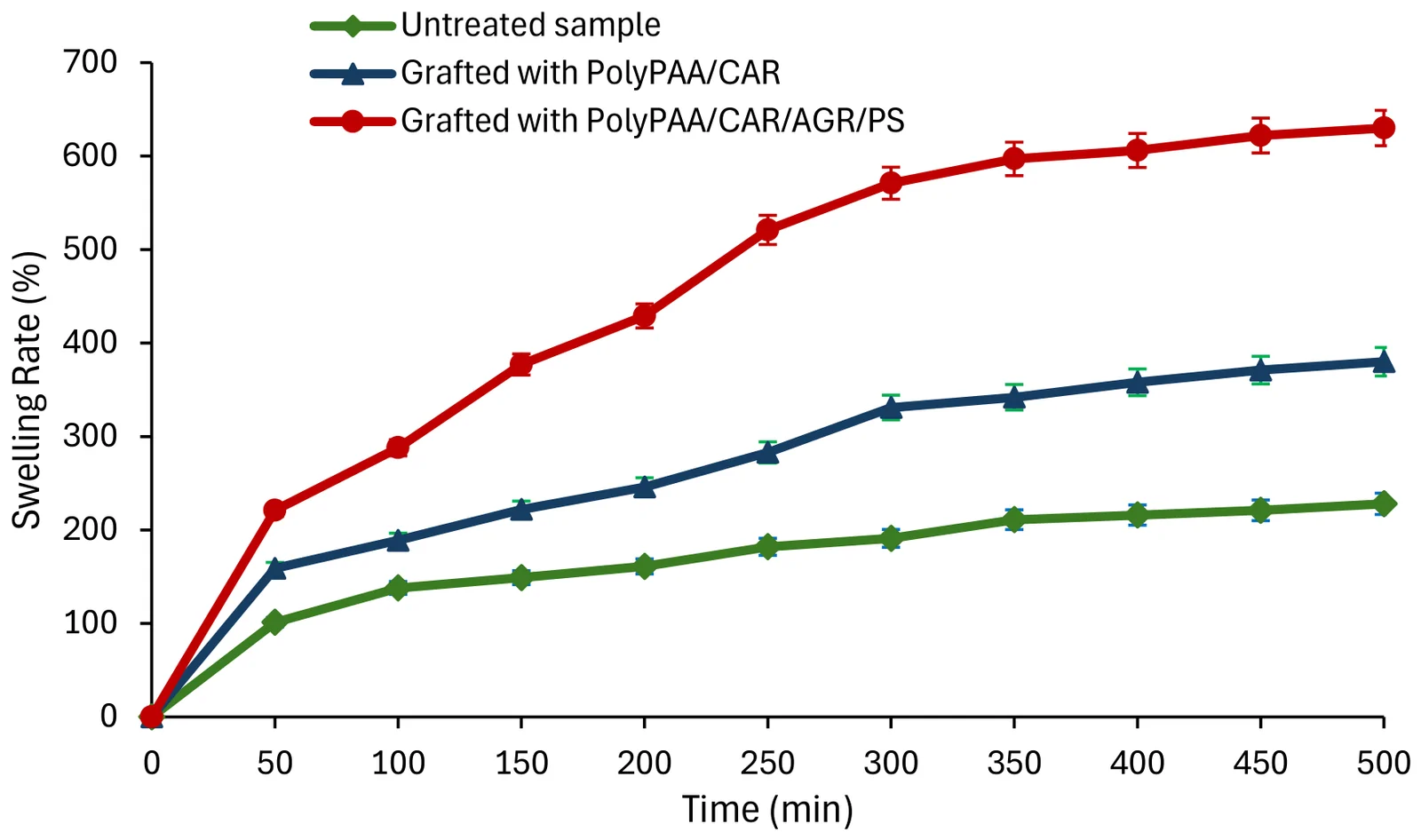
Comparison of swelling behavior between untreated and grafted cellulosic substrates functionalized with various polymer systems.

Water absorption capacity was evaluated to characterize the water uptake and swelling behavior of the untreated and functionalized dressings under aqueous conditions relevant to wound-dressing applications. As shown in Figure 4, the functionalized samples exhibited a higher swelling ratio than untreated cellulose, consistent with increased water uptake following treatment. Swelling increased over the first 300 min and subsequently approached a plateau, suggesting near-equilibrium water uptake under the tested conditions. The polyPAA/CAR/AGR/PS dressings exhibited the highest swelling ratio. However, because agar and propolis were introduced together and a compositionally matched propolis-free formulation was not included, their individual contributions cannot be distinguished. These results characterize the complete formulation and do not independently establish network integrity, fluid retention under pressure, or performance in wound exudate. The increased water uptake supports further investigation of fluid-handling properties for wound-dressing applications but does not demonstrate promotion of tissue repair.

### 3.3. FTIR Analysis

**Figure 5:**
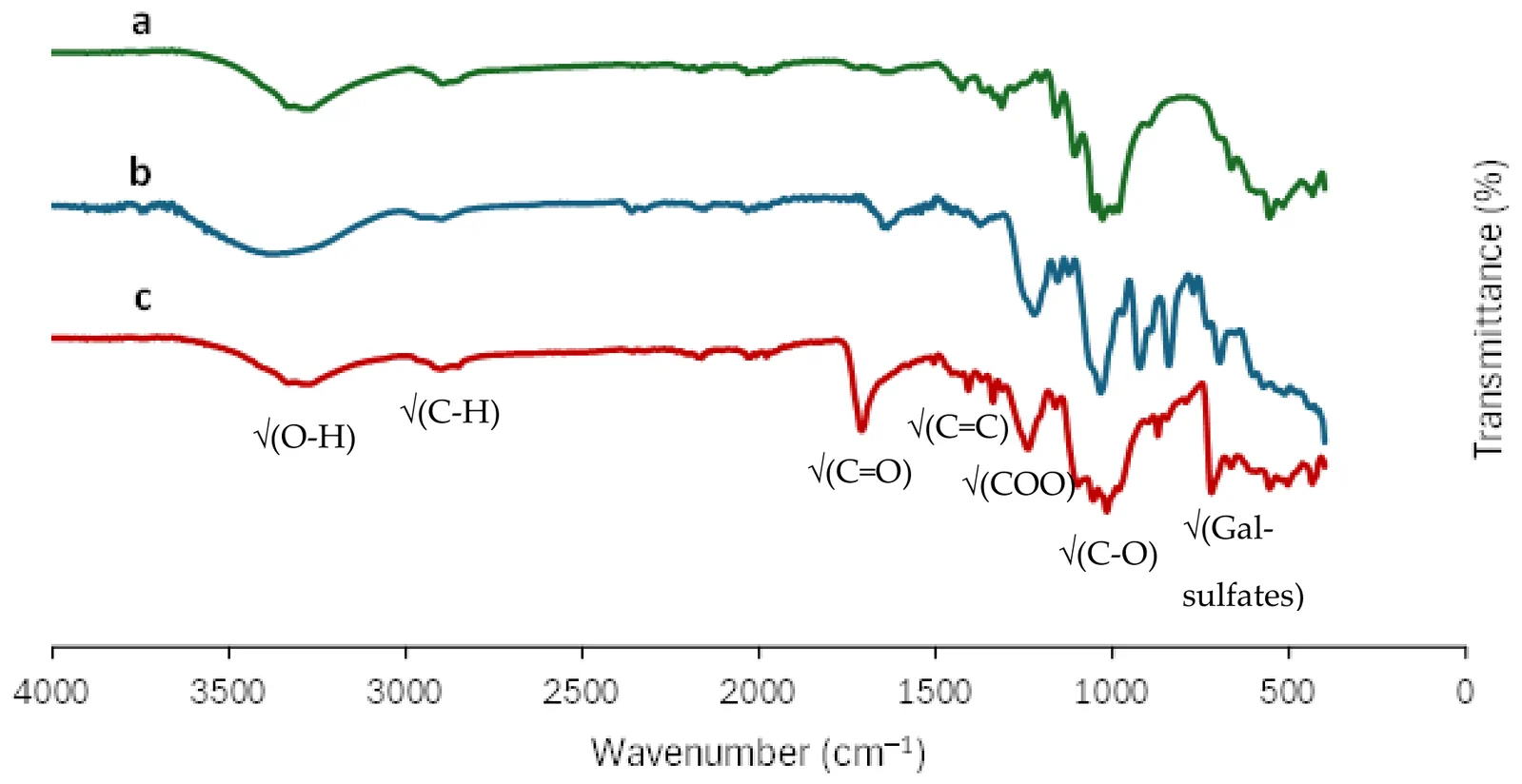
FT-IR spectra of (a, green) carrageenan, (b, blue) untreated cellulosic material, and (c, red) grafted cellulosic material.

The chemical changes of cellulose dressings after grafting with CAR/AGR and PAA were studied using ATR-FTIR spectroscopy (Figure 5). The spectra of untreated cellulose showed distinctive bands at about 3340 cm^−1^ (O–H stretching), 2900 cm^−1^ (C–H stretching), and 1050 cm^−1^ (C–O–C stretching of the cellulose backbone).

After functionalization, additional absorption bands appeared in the 1720–1735 cm^−1^ region, consistent with C=O stretching vibrations from carbonyl-containing species, potentially including ester groups, PAA carboxylic-acid groups, and propolis-derived constituents. This observation is compatible with, but does not independently establish, the proposed PAA-mediated esterification between the carboxyl groups of poly(acrylic acid) (PAA) and the hydroxyl groups of cellulose, κ-carrageenan, and agar. The overlapping contributions prevent this absorption region from being assigned uniquely to newly formed covalent ester linkages. The resistance of the functionalized samples to extensive washing with hot water suggests retention of deposited material under the applied washing conditions. However, this persistence does not distinguish covalent attachment from strong noncovalent interactions, physical entrapment, or insoluble deposits. These findings are compatible with mechanisms discussed in our previously published studies on related textile functionalization systems [19,22]. Taken together, ATR-FTIR and washing observations support treatment-associated chemical changes and material retention, while esterification and covalent crosslinking remain proposed mechanisms rather than experimentally verified bond formation in this multicomponent system.

Shifts and intensity changes in the O–H and C–O regions indicate differences in the chemical environment following treatment. These changes are compatible with the presence of deposited components and altered intermolecular interactions but do not independently demonstrate chemical linking of CAR, AGR, or propolis to cellulose. The spectra support functionalization-associated changes without establishing a unique bonding mechanism.

### 3.4. Mechanical Properties

The maximum tensile force of the dressings was evaluated to determine the effect of grafting on mechanical performance. The effect of polymer concentration on grafting mass gain and mechanical properties was evaluated using five concentration levels (untreated, C/4, C/2, 3C/4, and C), while maintaining constant curing conditions of 140 °C for 15 min. These conditions were selected to assess the influence of polymer concentration while minimizing additional thermal exposure that could adversely affect the original textile properties.

**Figure 6:**
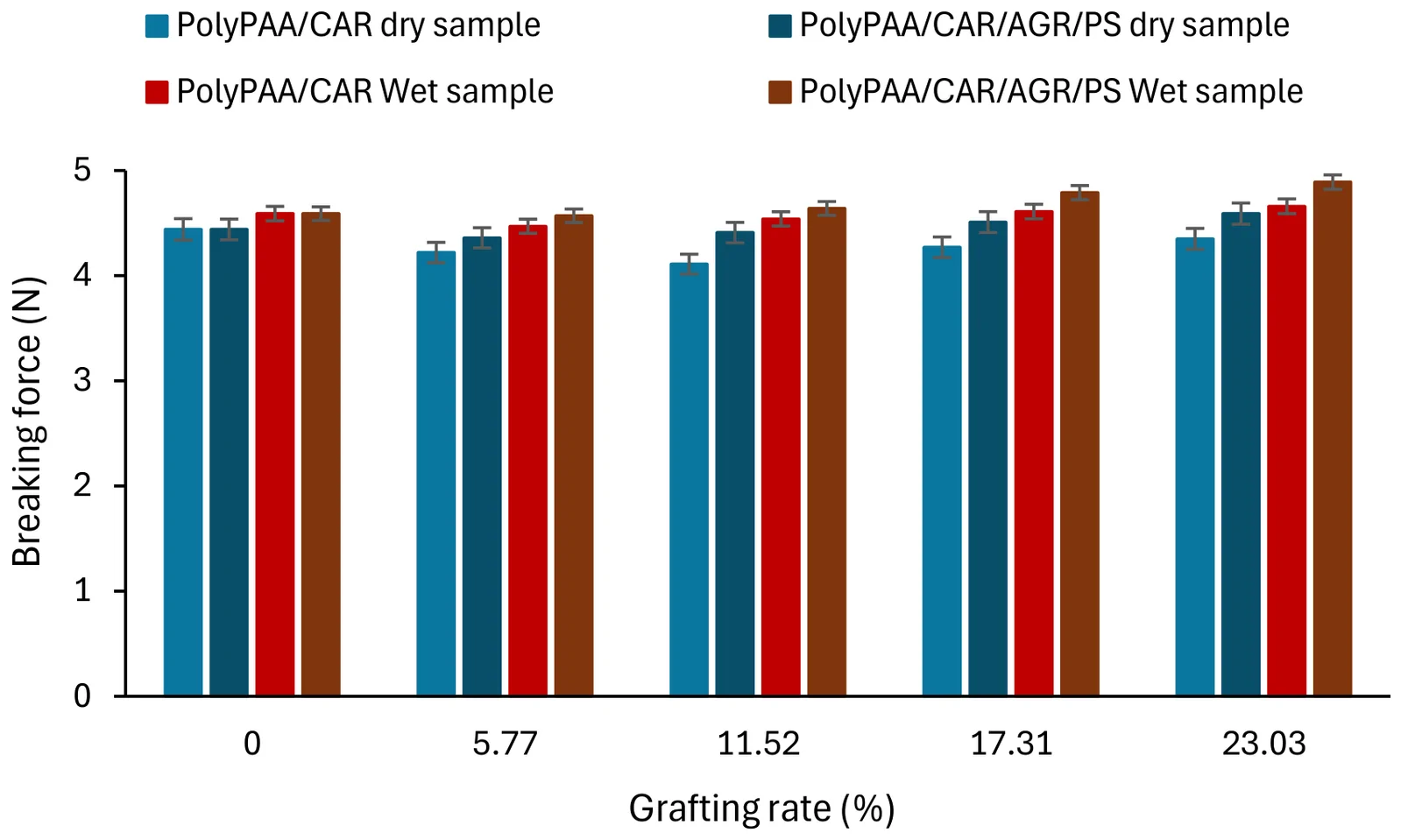
Breaking force of untreated and functionalized cellulosic textiles under dry and wet conditions, using formulation concentrations C/4, C/2, 3C/4, and C and a fixed curing treatment of 140 °C for 15 min. Results are expressed as mean ± SD for ten specimen replicates per condition.

As presented in Figure 6, functionalized dressings showed improved breaking force compared with untreated cellulose, consistent with a treatment-associated improvement in tensile performance, without independently establishing reinforcement through covalent crosslinking. Wet samples exhibited higher breaking force than dry samples under the tested conditions. Hydration may influence fiber swelling, yarn interactions, coating behavior, and load transfer; however, these contributions were not measured separately, and the observed increase cannot be attributed specifically to water-induced plasticization. The polyPAA/CAR/AGR/PS system demonstrated slightly higher breaking force than polyPAA/CAR. Because these formulations differ in both agar and propolis content, this difference cannot be assigned specifically to agar incorporation or enhanced network cohesion. The measurements demonstrate improved breaking force under the experimental conditions but do not establish suitability for clinical handling or application.

### 3.5. Surface Morphology (SEM Analysis)

**Figure 7:**
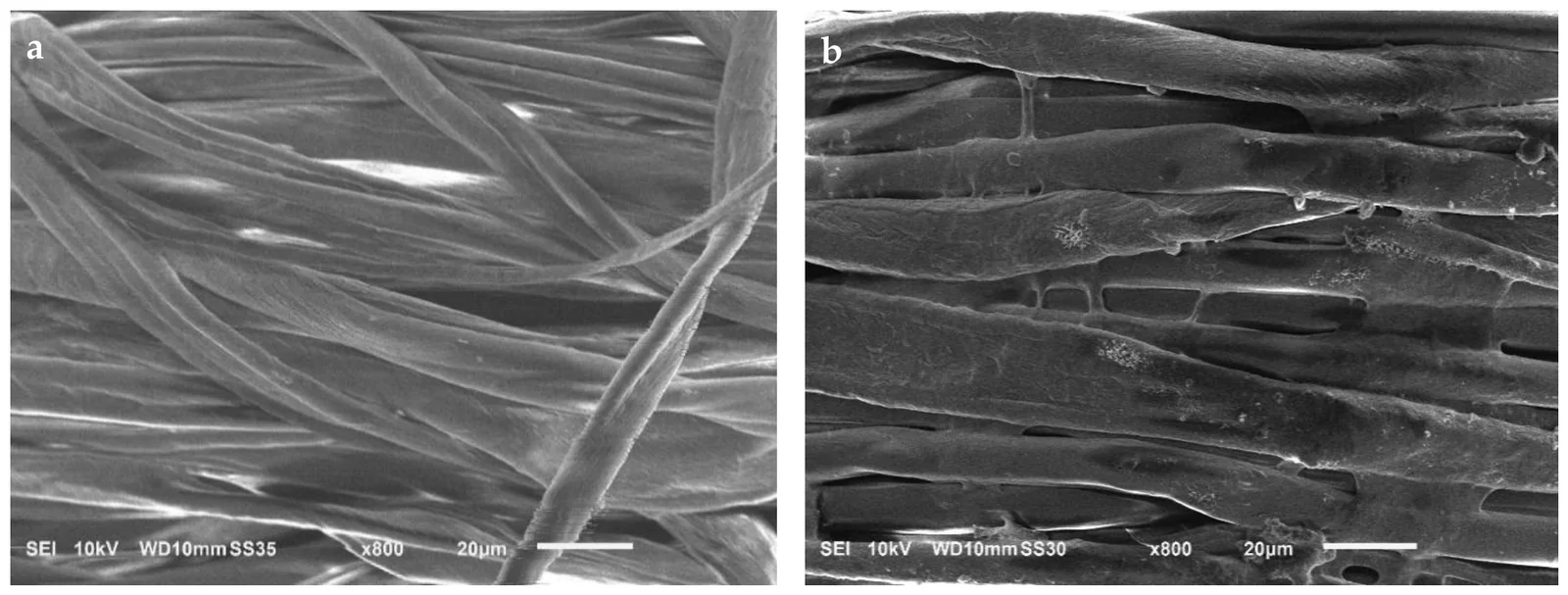
SEM analysis of surface morphology for untreated (**a**) and treated (**b**) cellulosic dressings.

SEM micrographs (Figure 7) revealed clear morphological differences between untreated and functionalized dressings. The untreated cellulose fibers showed smooth surfaces without a visible coating. In contrast, the functionalized sample displayed the presence of a polymeric coating with a slightly roughened surface texture, consistent with the formation of crosslinked CAR/AGR layers. Despite these surface modifications, the fibrous structure of the cellulosic substrate remained visibly preserved. No obvious polymer aggregates or substantial clogging of the inter-fiber spaces were observed. These findings suggest that the polymeric coating was distributed over the textile fibers without markedly obstructing the original inter-fiber structure.

### 3.6. Biological Evaluation

#### 3.6.1. Cell Viability

**Figure 8:**
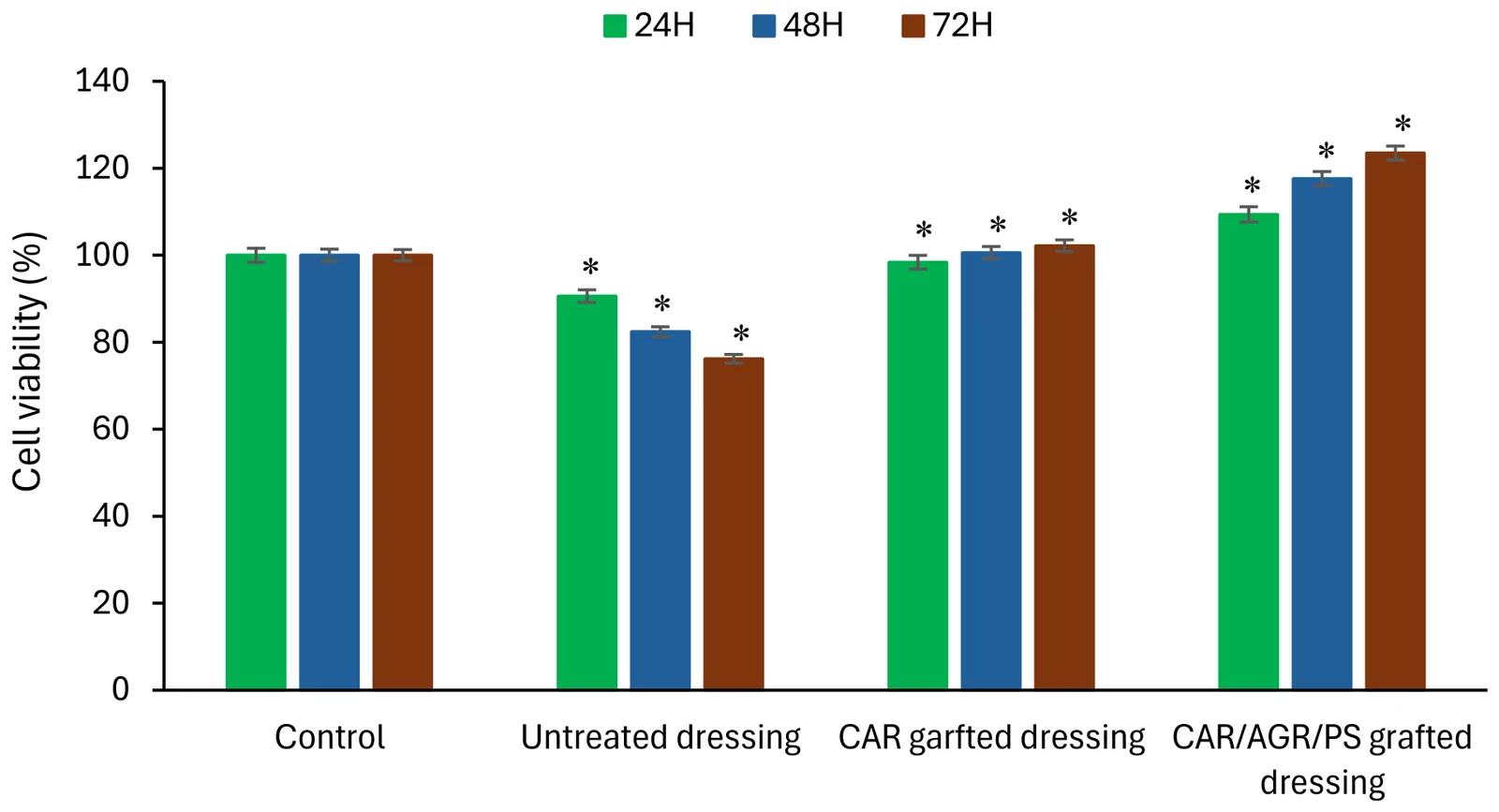
Apparent relative MTT response of HepG2 cells following exposure to untreated and functionalized cellulosic textiles, normalized to the corresponding cell-only control. The assay reflects metabolic reduction of MTT and does not directly measure proliferation. Results are expressed as mean ± SD from three separate experiments (*n* = 3), each with three technical repeats per condition averaged before analysis. Separate specimens were used at each time point. (*) indicates significant differences relative to the control (*p* < 0.05).

The in vitro cellular responses to the untreated and functionalized dressings were evaluated using the MTT assay with HepG2 cells (Figure 8). The untreated sample exhibited a decrease in cell viability with increasing contact duration, indicating a time-dependent reduction in cellular metabolic activity under the conditions tested. In contrast, the polyPAA/CAR-functionalized dressings exhibited cell viability comparable to that of the untreated cell control. The polyPAA/CAR/AGR/PS dressings showed apparent cell viability exceeding 100% after 24 h of exposure. Values exceeding 100% indicate a higher MTT assay signal relative to the cell-only control and do not independently demonstrate increased cell number or proliferation. Material-derived compounds may influence absorbance, tetrazolium reduction, or formazan recovery. Without verified material-specific interference controls, a contribution from noncellular effects cannot be excluded.

These findings provide a preliminary assessment of cellular responses to the developed dressings under the experimental conditions used. HepG2 cells, a human hepatocellular carcinoma-derived cell line, were used to assess cellular metabolic responses to the dressing materials. However, because these cells are not representative of the principal cell types involved in wound healing, the results cannot be directly extrapolated to skin-cell responses or wound-healing outcomes. MTT results alone are also insufficient to establish overall biocompatibility or suitability for wound-care applications. Further studies, including extract-based cytotoxicity testing, irritation, sensitization, hemocompatibility, inflammatory response, and evaluation using wound-relevant cell models, such as human dermal fibroblasts and keratinocytes, are required before conclusions regarding the safety of the developed dressings for wound applications can be drawn.

#### 3.6.2. Cell Adhesion Assessment

**Figure 9:**
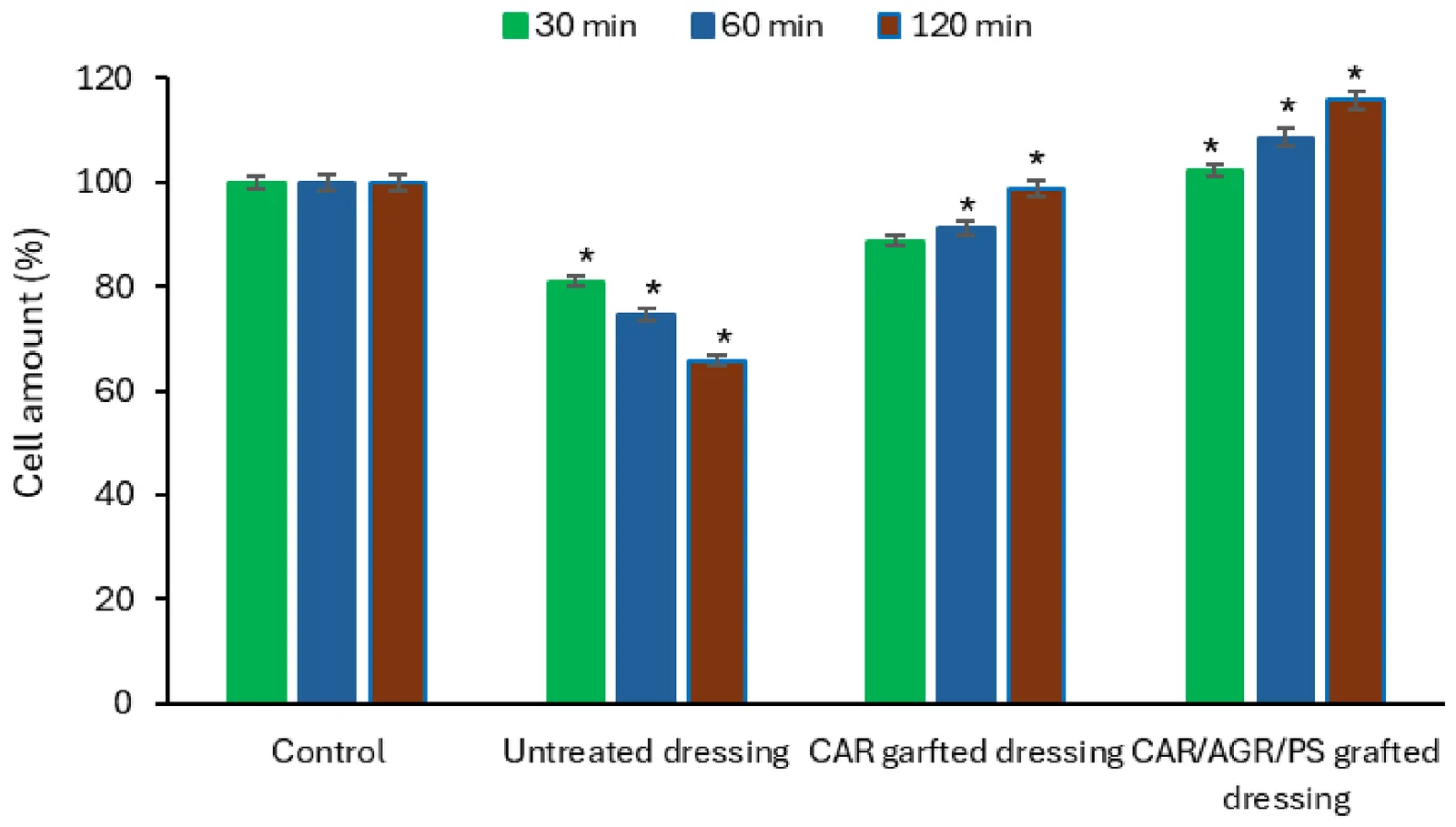
Cell adhesion of HepG2 epithelial cells on untreated, CAR-grafted, and polyPAA/CAR/AGR/PS-functionalized cellulosic dressings. Results are expressed as mean ± SD for ten specimen replicates per condition, with * indicating significant differences relative to the control (*p* < 0.05).

Functionalized dressings promote early cellular attachment in cell adhesion experiments. Figure 9 shows that the measured cell adhesion response for HepG2 cells increased with time and reached a maximum at 120 min. PolyPAA/CAR/AGR/PS samples showed somewhat better measured adhesion than polyPAA/CAR samples. This difference may be associated with changes in the surface properties following the incorporation of agar and propolis; however, the specific mechanisms responsible for the observed response were not investigated. These results suggest that the functionalized dressings supported early cellular attachment under the conditions tested. However, this preliminary observation does not establish overall biocompatibility or suitability for wound-healing applications, particularly because HepG2 cells are not representative of the principal cell types involved in wound healing. Further assessment using wound-relevant cells, such as human dermal fibroblasts and keratinocytes, is required.

### 3.7. Antimicrobial Activity

**Figure 10:**
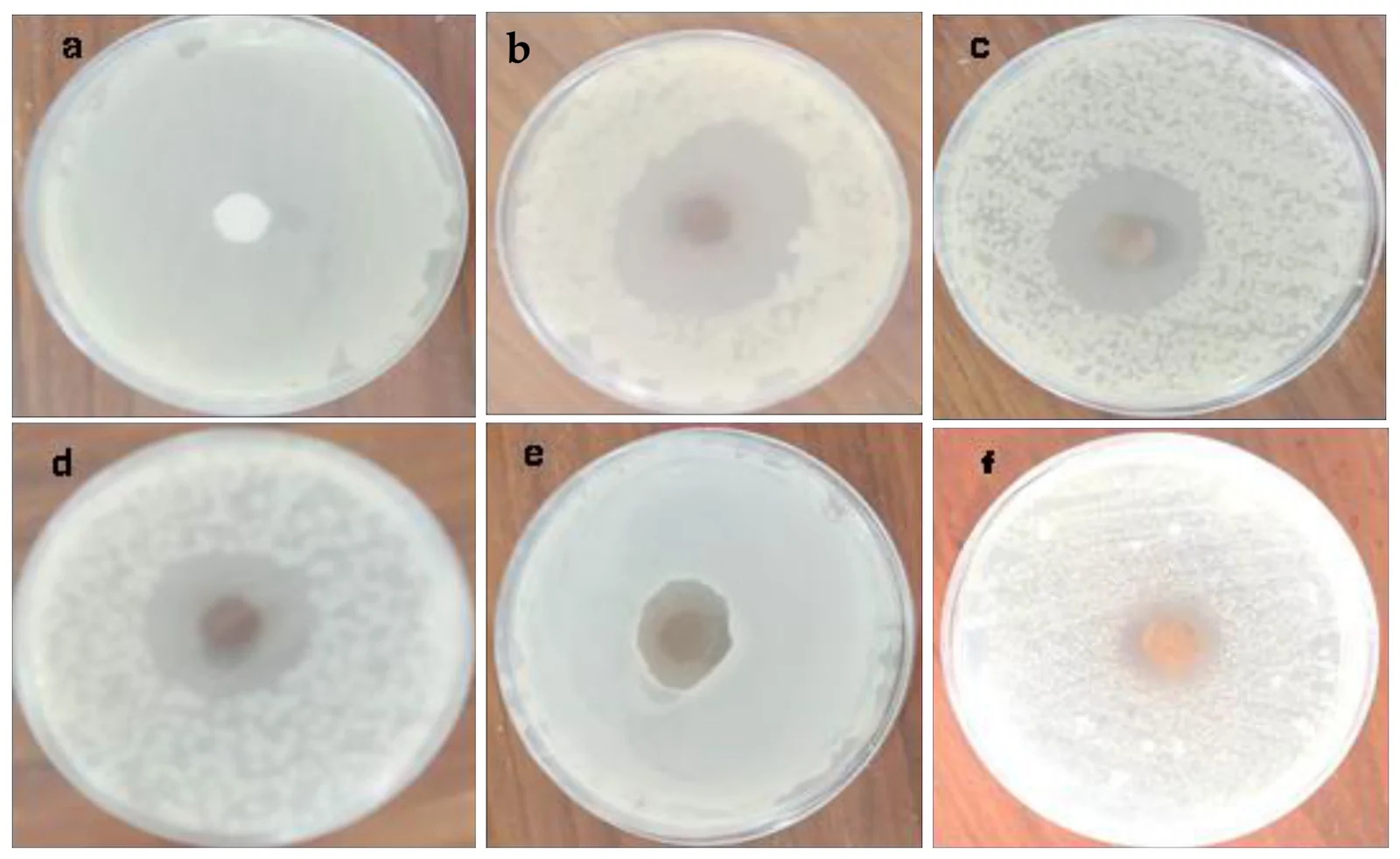
Microbiological screening of untreated sample against *S. aureus* (**a**) and polyPAA/CAR/AGR/PS-functionalized cellulosic dressings against *S. aureus* (**b**), *E. faecalis* (**c**), *E. coli* (**d**), *P. aeruginosa* (**e**), and *C. albicans* (**f**).

**Figure 11:**
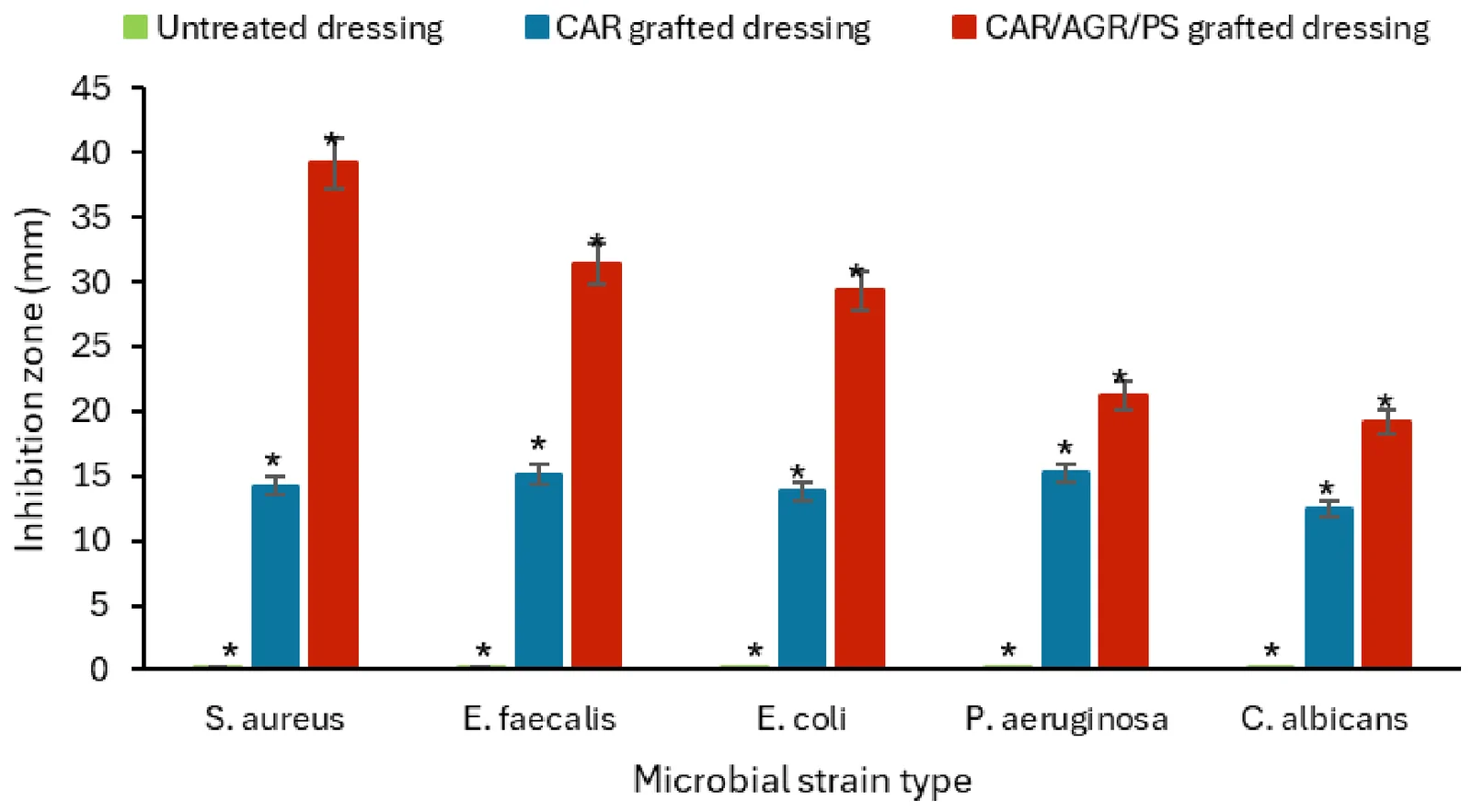
Antibacterial and antifungal activity of untreated and CAR/AGR/PS-functionalized cellulosic dressings against *S. aureus*, *E. faecalis*, *E. coli*, *P. aeruginosa*, and *C. albicans*. Data are mean ± SD (*n* = 3); * indicates *p* < 0.05 versus control. Functionalized dressings showed broad-spectrum activity.

The functionalized dressings inhibited the growth of all tested bacterial strains (Gram-positive *S. aureus* and *E. faecalis* and Gram-negative *E. coli* and *P. aeruginosa*) and *Candida albicans* compared with untreated cellulose (Figure 10 and Figure 11). These observations are associated with the complete treated formulation and cannot be attributed specifically to propolis or any other individual component. Although propolis has documented antimicrobial activity, the absence of a compositionally matched PAA/CAR/AGR formulation without propolis prevents its contribution from being distinguished from those of the polymeric matrix, residual processing reagents, or treatment-induced changes in surface chemistry. The PAA/CAR comparator differs from the complete formulation in both agar and propolis content and therefore does not isolate propolis-related effects. Synergistic interactions among the components were not demonstrated. Possible UV-induced changes in propolis-derived constituents and their release or activity were not independently assessed; consequently, the findings cannot be assumed to represent the activity of the material before UV exposure. The inhibition zones indicate that inhibitory substances associated with the treated dressings diffused into the surrounding agar and suppressed microbial growth under the experimental conditions; however, these results do not independently establish bactericidal or fungicidal activity. Further studies incorporating a compositionally matched propolis-free formulation, propolis-only, processing-reagent, and solvent controls, together with contact-kill or quantitative microbial log-reduction assays, are needed to clarify the contribution of each component and assess direct antimicrobial efficacy.

### 3.8. Anticancer Activity

**Figure 12:**
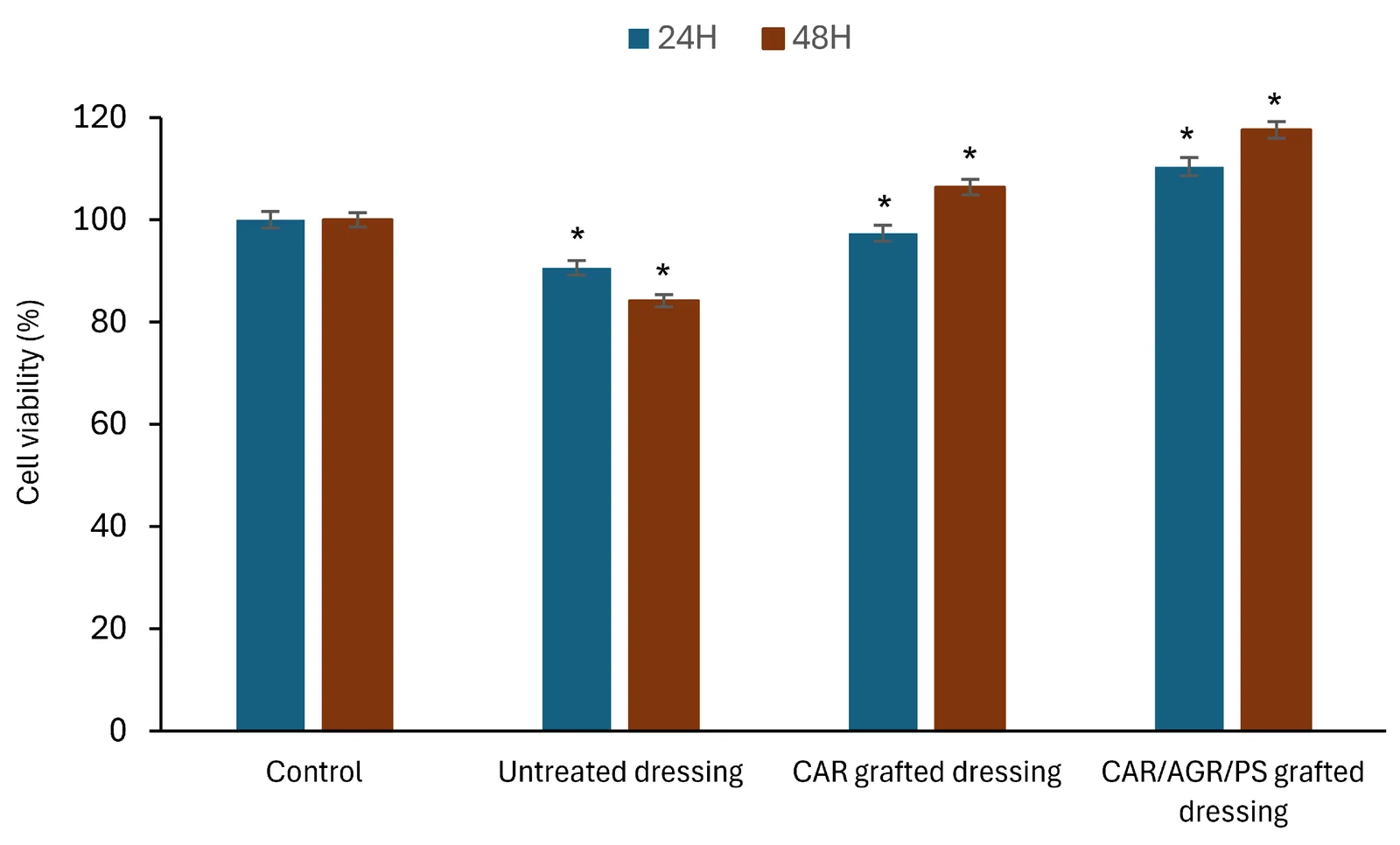
Cell viability of MCF10A (normal breast epithelial) cells after 24 and 48 h exposure to untreated, CAR-grafted, and CAR/AGR/PS-grafted cellulosic dressings. Data are shown as a percentage of the control (100%). Asterisks (*) represent *p* < 0.05. Results are expressed as mean ± SD from three separate experiments (*n* = 3), each with three technical replicates per condition averaged before analysis. Separate specimens were used at each time point.

**Figure 13:**
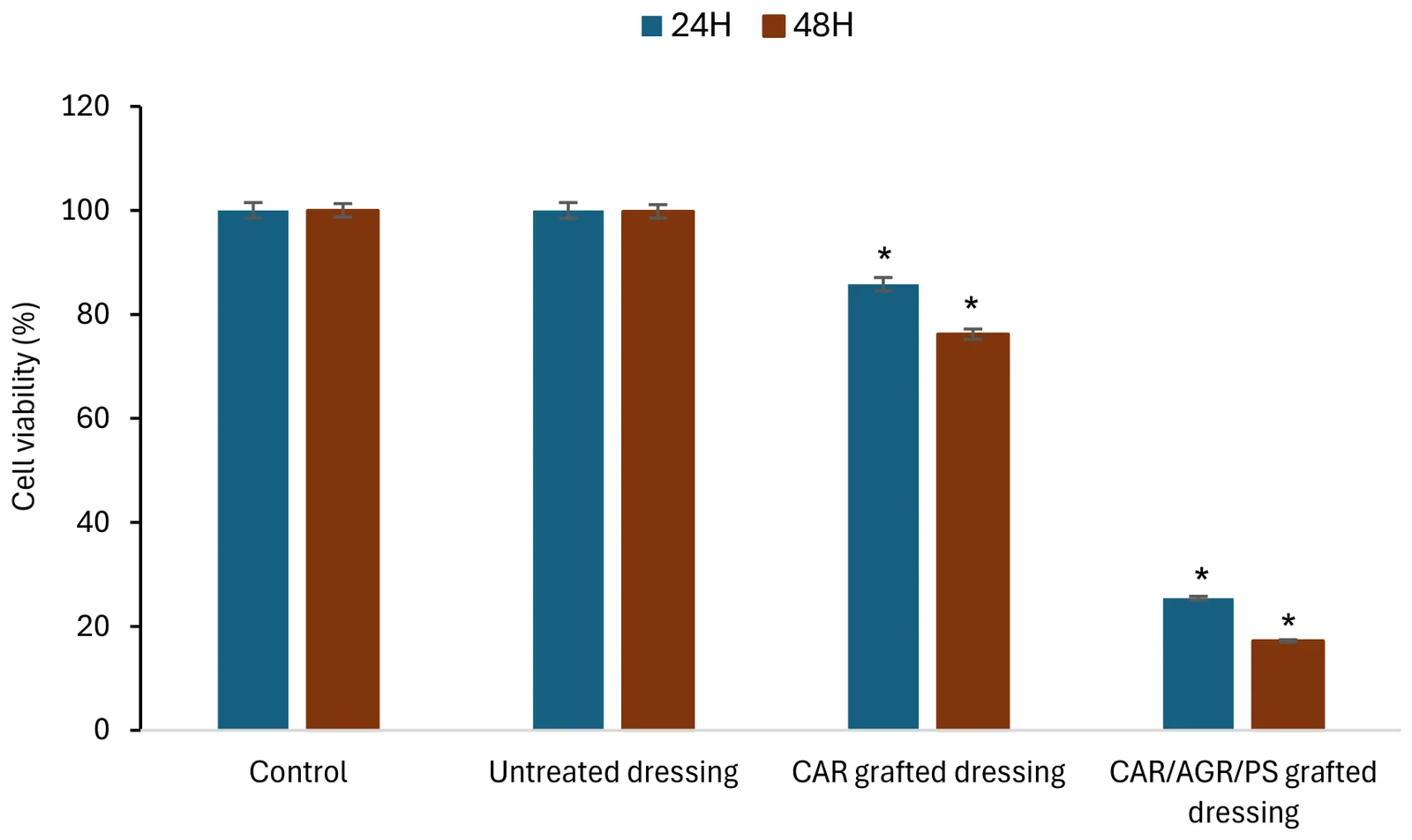
MTT assay-based cell viability of MCF-7 human breast cancer cells after 24 and 48 h exposure to untreated, CAR-grafted, and CAR/AGR/PS-grafted cellulosic dressings. Data are shown as a percentage of the control (100%). Asterisks (*) represent *p* < 0.05. Results are expressed as mean ± SD from three separate experiments (*n* = 3), each with three technical repeats per condition averaged before analysis. Separate specimens were used at each time point.

The effects of the polyPAA-CAR-AGR-PS dressings on cellular metabolic activity were evaluated using MTT assays with MCF-7 breast cancer cells and non-tumorigenic MCF10A breast epithelial cells (Figure 12 and Figure 13). Following exposure to the functionalized dressings, the MTT signal in MCF-7 cells decreased to 25.4% and 17.2% of the untreated control values after 24 and 48 h, respectively. In contrast, MCF10A cells showed comparatively limited changes under the tested conditions. These findings suggest differential cellular metabolic responses to exposure to the functionalized dressings. However, because the study did not include a dose–response assessment, apoptosis analysis, or propolis-release measurements, the mechanisms underlying these responses remain undetermined. Furthermore, the current experimental design does not distinguish effects associated with direct contact with the dressing from those caused by compounds released into the culture medium. Accordingly, these results should be interpreted as preliminary in vitro findings and do not, on their own, establish selective anticancer activity or therapeutic efficacy.

## 4. Discussion: Structure–Property–Bioactivity Relationships

A multifunctional cellulosic material was prepared by functionalizing a woven cellulose substrate with a PAA-crosslinked agar/κ-carrageenan biopolymer network and incorporating propolis into the formulation. The curing conditions (140 °C for 15 min) were selected based on the processing conditions reported in previous work [23,53] and adopted in the present study; no new response surface methodology (RSM) optimization was performed. ATR-FTIR analysis revealed spectral features consistent with carbonyl-containing structures, including possible ester linkages associated with the proposed PAA-mediated crosslinking mechanism. The persistence of the polymeric coating after the washing procedure, together with the appearance of new FTIR absorption features consistent with ester formation, provides complementary evidence supporting the retention and chemical modification of the coating on the cellulosic substrate following PAA/SHP-mediated treatment. The persistence of the coating suggests effective retention under the applied washing conditions, while the spectroscopic changes are consistent with possible esterification involving carboxyl and hydroxyl groups. Although these findings support the proposed functionalization and crosslinking mechanism, they do not independently establish the precise distribution or extent of covalent linkages within the resulting material. In particular, the absorption band in the 1720–1735 cm^−1^ region cannot be regarded as conclusive evidence of covalent grafting because PAA and propolis may also contribute to the carbonyl absorption in this region. The available results also do not establish covalent attachment of propolis to cellulose.

SEM morphological analysis revealed morphological changes consistent with the presence of a polymeric coating on the cellulose fibers. The functionalized materials showed higher water uptake and changes in mechanical characteristics in dry and wet conditions, pointing to the role of the crosslinked biopolymer network in the physicochemical performance of the cellulose substrate. Although the observed changes in swelling, mechanical response, and biological assay signals were associated with the functionalized formulations, the present study does not independently isolate the contribution of each component. In particular, the absence of a compositionally matched PAA–CAR–AGR control without propolis, together with the lack of quantitative propolis loading and release data, limits the attribution of specific biological effects to propolis. ATR-FTIR and SEM provide supporting evidence of chemical and morphological changes, but they do not independently establish covalent attachment of every component or demonstrate unchanged porosity. Further work should include appropriate formulation controls, quantitative surface and chemical analyses, residual-chemical assessment, propolis release measurements, and testing with wound-relevant cellular models. In addition, quantitative assessment of porosity and evaluation of the material under physiologically relevant fluid conditions would be needed to establish its structural and fluid-handling performance for wound-care applications.

The biological examination also indicated cellular metabolic responses in the HepG2 MTT assay and cell adhesion in the separate adhesion assessment, besides antibacterial activity against the tested Gram-positive and Gram-negative bacteria and antifungal activity against C. albicans. The propolis-containing materials also exhibited differential cellular viability responses against MCF-7 cells in comparison to MCF10A cells. Taken together, these results indicate that functionalization with a crosslinked agar/κ-carrageenan network and incorporation of propolis were associated with changes in the physicochemical and biological responses of the cellulosic substrate under the conditions tested. Comparatively, Table 2 summarizes these findings alongside results reported for previously described polysaccharide-functionalized cellulosic materials. However, direct comparisons should be interpreted cautiously because differences in material composition, experimental protocols, and test conditions may influence the reported values. Thus, the resulting material provides a promising platform for further development and biological validation of bioactive polymeric wound-dressing materials, rather than demonstrating established clinical suitability. Future studies should focus on long-term stability, release behavior, biocompatibility using wound-relevant cell models, comprehensive wound-dressing performance, and in vivo wound-healing performance.

The various chemical functionalities of κ-carrageenan and agar may potentially contribute differently to the structure and hydration of the grafted layer. κ-Carrageenan has sulfate groups that confer anionic character and can form electrostatic and hydrogen-bonding interactions, while agar contains numerous hydroxyl functions that may be involved in hydrogen bonding within the polymer network. These qualities can assist in water retention and network organization. However, although these structural features may influence the biological response of the material, the present work did not explicitly measure surface charge or the molecular interactions responsible for antibacterial activity. Hence, the biological role of the polysaccharide components should be seen as a possible structure–property link and not as an explicitly proven antibacterial mechanism.

The incorporation of propolis provides a potential source of additional biological functionality to the polymeric system. Propolis is a chemically complex natural product containing phenolic acids, flavonoids, and other bioactive constituents, whose composition varies considerably depending on its botanical and geographical origin [46,47,48]. Propolis has been associated with broad-spectrum antimicrobial activity through several proposed mechanisms, including disruption of microbial membrane integrity and permeability, interference with energy metabolism, and inhibition of cellular functions [49,50]. These literature-reported mechanisms provide background context but were not demonstrated for the present formulations. Furthermore, the composition and quantity of propolis-derived compounds retained after drying, thermal curing, and washing were not determined. The nominal extract concentration in the impregnation bath therefore cannot be equated with the propolis content of the finished textile, and the measured mass gain does not quantify propolis loading. Propolis loading efficiency, thermal stability during processing, and release behavior from the functionalized textile were also not experimentally determined. Consequently, preservation of the initial extract composition and its original biological activity cannot be assumed, and the observed biological responses cannot be related to a measured dose of propolis or individual phenolic constituents. These limitations should be addressed through quantitative extract characterization, analysis of retained chemical markers, thermal characterization, and release measurements to clarify the relationships between formulation, processing, and antimicrobial performance. Moreover, because a compositionally matched propolis-free PAA–CAR–AGR control was not included, the specific contribution of propolis cannot be separated conclusively from the effects of the polymer network and other formulation constituents.

Furthermore, propolis and its phenolic constituents have been reported to exert cytotoxic and antiproliferative effects in several cancer-cell models, including MCF-7 cells [53,54]. These effects have been associated in previous studies with mechanisms such as oxidative stress, apoptosis, cell-cycle regulation, and modulation of intracellular signaling pathways. However, these mechanisms were not directly investigated in the present study. MCF-7 cultures exhibited lower normalized MTT responses than MCF10A cultures under their respective assay conditions, indicating different assay responses to the complete propolis-containing formulation. These differences do not independently establish cell death, apoptosis, selective anticancer activity, or a propolis-specific mechanism. Differences between the cell models and culture media, together with possible material-related optical or chemical interference with the MTT assay, limit direct comparisons. Accordingly, the findings are interpreted as differential metabolic assay responses rather than demonstrated differential cytotoxicity or an anticancer effect. Further studies using complementary assays, appropriate dose–response experiments, and additional cancerous and non-tumorigenic cell models are required to investigate the underlying mechanisms and determine whether the observed differences reflect genuine cell-type-selective effects [55,56,57]. In addition, a ratio of normalized MTT responses between MCF10A and MCF-7 cells at a single exposure condition is a relative assay-response ratio, not a conventional selectivity index based on IC_50_ values.

Overall, the results suggest formulation-associated relationships between material properties and biological assay responses, rather than demonstrated causal effects of individual constituents. The proposed roles of PAA-mediated interactions, agar and κ-carrageenan, and propolis-derived compounds remain hypotheses because compositionally matched controls were not included. The combined formulation resulted in a functionalized cellulosic material with increased water uptake, improved breaking force relative to untreated cellulose under the tested conditions, and measurable biological responses, including antibacterial and antifungal activity and differential MTT responses. These observations concern the complete treated formulation and do not establish propolis-specific effects or synergy among its components. The MTT findings obtained with HepG2 cells provide a preliminary assessment of cellular metabolic responses but cannot establish cytocompatibility for wound-care applications because HepG2 cells are not representative of the principal cell types involved in wound healing. Potential material-related interference with the MTT assay also cannot be excluded. Likewise, the cell-adhesion findings should be interpreted in accordance with the verified identity of the cells used in that experiment. The present findings thus provide a basis for further investigation of the relationships between formulation, structure, physicochemical properties, and biological responses, while further studies of propolis release, surface chemistry, long-term stability, wound-relevant cell responses, and in vivo performance are needed to establish its suitability for practical wound-dressing applications. Additional assessments of water-vapor transmission, fluid retention under pressure, wet-state integrity, degradation behavior, and broader biocompatibility are also required before practical wound-dressing suitability can be concluded.

## 5. Conclusions

A multifunctional cellulosic material was developed by functionalizing a cellulose substrate with a PAA-crosslinked agar/κ-carrageenan biopolymer and integrating propolis as a bioactive component. The optimal curing setting was 140 °C for 15 min according to response surface optimization. ATR-FTIR analysis provided evidence consistent with ester-containing species and the proposed PAA-mediated esterification and crosslinking mechanism, whereas SEM revealed the formation of a polymeric coating on the cellulose fibers; however, its effect on porosity was not established. The functionalized materials showed higher water uptake and improved tensile characteristics under dry and wet conditions, pointing to the potential contribution of the crosslinked biopolymer network in the physicochemical performance of the cellulose substrate.

The biological analysis further indicated high metabolic viability of HepG2 cells under the tested conditions, along with increased cell adhesion, besides antibacterial activity against the tested Gram-positive and Gram-negative bacteria and antifungal activity against *C. albicans*. The propolis-containing materials also exhibited differential cellular metabolic responses in MCF-7 cells in comparison to MCF10A cells. Collectively, these results indicate that the functionalized cellulose materials exhibit distinct physicochemical and biological properties. Thus, the resulting material provides a promising platform for further development as a candidate bioactive wound-dressing material, especially for future studies on long-term stability, release behavior, biocompatibility using wound-relevant cell models, and in vivo wound-healing performance.

## Figures and Tables

**Table 1:** Initial impregnating-solution concentration levels and corresponding grafting rates.

Sample	Relative Concentration	Mass Gain (%)
Untreated	No impregnating solution	0.00
C/4	25% of C	5.77
C/2	50% of C	11.52
3C/4	75% of C	17.31
C	100% of C	23.03

**Table 2:** Comparison of grafting mass gain, swelling behavior, mechanical properties, antimicrobial activity, and cell-viability responses of the present material with previously reported polysaccharide-functionalized cellulosic wound dressings.

Propriety	Present Study	Previous Study 1 [19]	Previous Study 2 [21]
Polysaccharide used	Agar/κ-carrageenan	Chitosan/Suaeda fruticosa polysaccharide extract	Alginate/Carthamus tinctorius polysaccharide extract
Mass gain (%)	23.03	19.4	22.8
Swelling (%)	630	421	Not reported
Wet breaking force (N)	4.89	4.52	4.21
Antimicrobial inhibition zone (mm) (S. a/E. c)	39.2–29.3	24.1/22.4	25.5/20.6
Apparent relative HepG2 MTT response at 72 h (%)	132.5	85.2	122.2

Abbreviations: S. a, *Staphylococcus aureus*; E. c, *Escherichia coli*.

## Data Availability

The data are contained within the article.
